# Building a Semantic Health Data Warehouse in the Context of Clinical Trials: Development and Usability Study

**DOI:** 10.2196/13917

**Published:** 2019-12-20

**Authors:** Romain Lelong, Lina F Soualmia, Julien Grosjean, Mehdi Taalba, Stéfan J Darmoni

**Affiliations:** 1 Department of Biomedical Informatics Rouen University Hospital Rouen France; 2 LITIS EA 4108 TIBS Normandy University Rouen France; 3 LIMICS U1142 Inserm Sorbonne University Paris France

**Keywords:** data warehousing, search engine, semantics, clinical trial, patient selection

## Abstract

**Background:**

The huge amount of clinical, administrative, and demographic data recorded and maintained by hospitals can be consistently aggregated into health data warehouses with a uniform data model. In 2017, Rouen University Hospital (RUH) initiated the design of a semantic health data warehouse enabling both semantic description and retrieval of health information.

**Objective:**

This study aimed to present a proof of concept of this semantic health data warehouse, based on the data of 250,000 patients from RUH, and to assess its ability to assist health professionals in prescreening eligible patients in a clinical trials context.

**Methods:**

The semantic health data warehouse relies on 3 distinct semantic layers: (1) a terminology and ontology portal, (2) a semantic annotator, and (3) a semantic search engine and NoSQL (not only structured query language) layer to enhance data access performances. The system adopts an entity-centered vision that provides generic search capabilities able to express data requirements in terms of the whole set of interconnected conceptual entities that compose health information.

**Results:**

We assessed the ability of the system to assist the search for 95 inclusion and exclusion criteria originating from 5 randomly chosen clinical trials from RUH. The system succeeded in fully automating 39% (29/74) of the criteria and was efficiently used as a prescreening tool for 73% (54/74) of them. Furthermore, the targeted sources of information and the search engine–related or data-related limitations that could explain the results for each criterion were also observed.

**Conclusions:**

The entity-centered vision contrasts with the usual patient-centered vision adopted by existing systems. It enables more genericity in the information retrieval process. It also allows to fully exploit the semantic description of health information. Despite their semantic annotation, searching within clinical narratives remained the major challenge of the system. A finer annotation of the clinical texts and the addition of specific functionalities would significantly improve the results. The semantic aspect of the system combined with its generic entity-centered vision enables the processing of a large range of clinical questions. However, an important part of health information remains in clinical narratives, and we are currently investigating novel approaches (deep learning) to enhance the semantic annotation of those unstructured data.

## Introduction

### Background and Significance

Hospitals maintain important health data that can be used in various contexts: first and foremost, clinical care and then data reusability, clinical decision support systems [1], clinical research and cohort selection [2], education [3,4], and indicators. However, the exploitation of these data remains difficult for several reasons. First, the data are produced and maintained by different systems and health professionals and are consequently spread over multiple sources and even across multiple establishments. Second, the significant amount of data generated results in problematic management of data both in terms of data storage capabilities and data access performances. Health data can synthetically and legitimately be described as *big data*. For instance, according to research [5,6], in the United States, the health care system alone reached 150 exabytes (1.5×10^20^ bytes) in 2011 and will reach the yottabyte scale (10^24^ bytes) in the near future. Moreover, the health data produced are of different nature; some data are natively structured (eg, diagnosis-related group [DRG] codings and laboratory tests results), but an important part of medical information remains in unstructured free-text clinical narratives (CNs; eg, admission notes, history and physical reports, discharge summaries, radiology reports, and pathology reports) [7]. This unstructured information is particularly relevant in the context of cohort selection tasks. However, in the study by Raghavan et al [8], the authors found that not only unstructured data were essential to resolve between 59% and 77% of some clinical trials criteria but also that combining the use of structured and unstructured data enabled leverage of patient recruitment. To process unstructured data, the main approaches rely on natural language processing (NLP) methods [9,10]. The background knowledge, as represented in terminologies and ontologies (T&Os; that describe the domain), plays a crucial role in any clinical NLP task [11]. A common approach to information retrieval (IR) in clinical unstructured text outside the basic full-text search comprises partially restructuring the original texts using semantic annotators (eg, MetaMap [12]) that map words or expressions to concepts from domain knowledge databases.

Consistently aggregating all these scattered, big, complex, and diversely structured data is, in fact, the role of health data warehouses (HDWs). An HDW is defined as a grouping of data from diverse sources accessible by a single data management system [13]. This kind of data repository centralizes clinical, demographic, and administrative data within a uniform and consistent data model. Many HDWs have been proposed worldwide. From a holistic point of view, the majority of these solutions provide aggregated data mainly focusing on patient data as a result. Furthermore, they do not necessarily allow the full and independent visualization and retrieval of the different atomic entities conceptually composing the whole scope of clinical information (eg, Stanford Translational Research Integrated Database Environment or STRIDE [14] and Data Warehouse for Translational Research or DW4TR [15]). This is, nevertheless, particularly important in an IR context, as potential clinical questions and inquiries from health professionals are formulated in terms of their vision of the conceptual organization of data that derive from the actual patient management process. The Enterprise Data Trust [16] relies heavily on industrial solutions to cope with the huge amount of data. Many solutions also implement generic frameworks, such as Informatics for Integrating Biology and the Bedside (i2b2) database. This, however, implies concessions to conciliate the original conceptual representation of data with the data model required by the framework (eg, The European Hospital George Pompidou HDW [17]). Furthermore, many standardized controlled vocabularies used to semantically describe health information do not always provide access to concepts in French, and access to the data through these T&Os is not always provided for the whole set of data notably as far as the unstructured data are concerned (eg, Electronic Medical Record Search Engine or EMERSE [18] and STRIDE [14]).

In this context, in 2017, the Biomedical Informatics and Information Department of Rouen University Hospital (RUH) initiated the conception and development of a semantic HDW (SHDW). The SHDW functionally relies on 3 independent semantic layers: layer 1—the cross-terminological health T&O portal (HeTOP) [19] that provides the background knowledge necessary to semantically describe the health data; layer 2—a semantic annotator, the extracting concepts from multiple terminologies (ECMT) [20,21], that enables the annotation of unstructured data; and layer 3, the semantic search engine (SSE) [22-24] and a Web application interface semantic access to health information, ASIS, that enable access and retrieval of health data through different conceptual entities composing health information. A generic entity-attribute-value (EAV) data model and a not only structured query language (NoSQL) layer (layer 0) enable data structuring while preserving the original conceptual data model.

This study aimed to present a proof of concept (POC) of this SHDW based on the data of 250,000 patients from RUH and to assess its ability to assist health professionals in prescreening eligible patients in a clinical trial context. Since November 2018, this POC has integrated all the data of 1.8 million patients from RUH.

### Related Studies

Clinical data warehousing manages health data from hospitals and is a well-addressed research field. Few generic frameworks and components exist. i2b2 [25,26] is a data mart used in >200 hospitals worldwide. Initiated within the Massachusetts General Hospital in 2004, i2b2 was developed by the Harvard Medical School and is funded by the National Institutes of Health. It enables the integration of clinical and genomic data into an EAV model known as the star schema. i2b2 enables the retrieval of patients’ data using graphically built queries and querying of free-texts and coded information. Another example of a distributed solution is the Observational Medical Outcomes Partnership Common Data Model [27]. This EAV model tends to standardize data from HDW at structure and representation levels (ie, terminologies and vocabularies).

In France, a few open-source solutions exist, such as Dr Warehouse [28]—the CN-oriented data warehouse of Necker Children’s Hospital, but 2 solutions really stand out from the others: the ConSoRe system [29] used in some French Oncology Hospitals and the query engine Biomedical data warehouse of the hospital whose French acronym is eHOP [30,31] that is being deployed in 6 University Hospitals in Western France.

Owing to the specificity of the data and their private and sensitive aspect, HDWs are specific systems that are used locally in Hospital Information Systems (HISs) rather than distributed and ready-to-use solutions, and many specific HDWs have been developed worldwide in addition to the previously cited generic solutions.

The STRIDE (United States) [14] project focuses on a clinical data warehouse supporting clinical and translational research. It was initiated in 2003 at Stanford University when the functionalities of i2b2 and CAncer Biomedical Informatics Grid were not considered optimal. An Oracle database and an EAV data model derived from the Health Level 7 Reference Information Model (RIM) standard are used for data storage and representation. Several (mainly English) standardized terminologies are used to represent important biomedical concepts and their relationships (eg, Systematized Nomenclature Of MEDicine–Clinical Terms or SNOMED-CT, RxNorm, 9th revision of the International Classification of Diseases or ICD-9–Clinical Modification, and current procedural terminology). STRIDE provides hierarchical concept-based retrieval as far as structured data are concerned and provides full-text search access to more than 6 million CNs. The system is based on an n-tiered architecture and the querying of the data is distributed along several client applications whose scope targets patient cohort selection, cohort chart review, clinical data extraction, research data management, and specimen data management. The querying is done graphically using drag and drop interface-based components and returns aggregated data as a result without exposing individual patient data.

EMERSE (Michigan, United States) [18] is an electronic health record–oriented system exclusively providing full-text search capabilities into free-text clinical notes.

The Windber Research Institute (United States) developed the DW4TR [15] system to support multiple translational research projects through highly structured medical information represented in 3 dimensions (namely, clinical data, molecular data, and temporal information). Data are collected into an Oracle Relational DataBase Management System (RDBMS) with an EAV data model and are subsequently hosted in an extensible data model that organizes it into a structure of hierarchical modules inherited from especially developed ontologies. It provides 2 graphical querying interfaces designed to provide aggregated data dedicated to data analysis (eg, mean, standard deviation, counts, categorical data, and chronological view).

The Enterprise Data Trust [16] is an industrial HDW initiated in 2005 at the Mayo Clinic (50,000 employees, Rochester, Minnesota, United States). It collects patient care, education, research, and administrative data to support IR, business intelligence, and high-level decision making. The Enterprise Data Trust strongly relies on industrial technologies (eg, InfoSphere Information Server—International Business Machines; iSight and iGuard—Teleran; BusinessObjects—Systems Applications and Products; and PowerDesigner—Sybase) and enables integration and exploitation of important volumes of data (eg, more than 7 million unique patients, 64 million diagnoses, and 268 million test results). The architecture and functionalities of the Enterprise Data Trust rely on legacy technical components and long-standing governance works on data and metadata management, data modeling, and standardized vocabularies. Those initiatives provide the HDW with a reliable organization of information on patient, genomic, and research data as well as querying capabilities for cohort selection and aggregate retrieval.

In 2008, the European Hospital George Pompidou (Paris, France) initiated an HDW [17] based on the i2b2 framework. It is strongly integrated in the clinical information system (IS) of the hospital that relies on several industrial solutions (eg, OneCall—McKesson; Act management, computerized physician order entry—Medasys; and integration platform—Thales). The core HDW infrastructure relies on an Oracle database for storage (1.2 million patients and 1 million stays) and the i2b2 framework for data representation. Several client applications are connected to the system to provide technical access to the data but mainly use i2b2 client as far as researchers are concerned. The SMart Eye DATabase (SMEYEDAT) [32] is an ophthalmologic-specialized HDW developed at the University Eye Hospital in Munich in Germany. SMEYEDAT is based on a Microsoft structured query language (SQL) database updated daily from the HIS and uses a star-like patient-centered data model for data representation. The QlikView (QlikTech) [33] tool was implemented as an analytic tool to visualize and explore patient data. This interface enables patient selection using criteria and views specific to the domain.

## Methods

Overall, the first prerequisite pertaining to the design of an HDW-based system is the extraction of data from the HIS. This can be achieved in 2 ways: by (1) setting up a data stream from the production environment (or a replicated database) to the HDW data storage component, or (2) using extract-transform-load (ETL) scripts. As far as the SHDW is concerned, ETL scripts are used. The following section describes the targeted sources of data of the HIS of RUH.

### Data Sources

**Figure 1:**
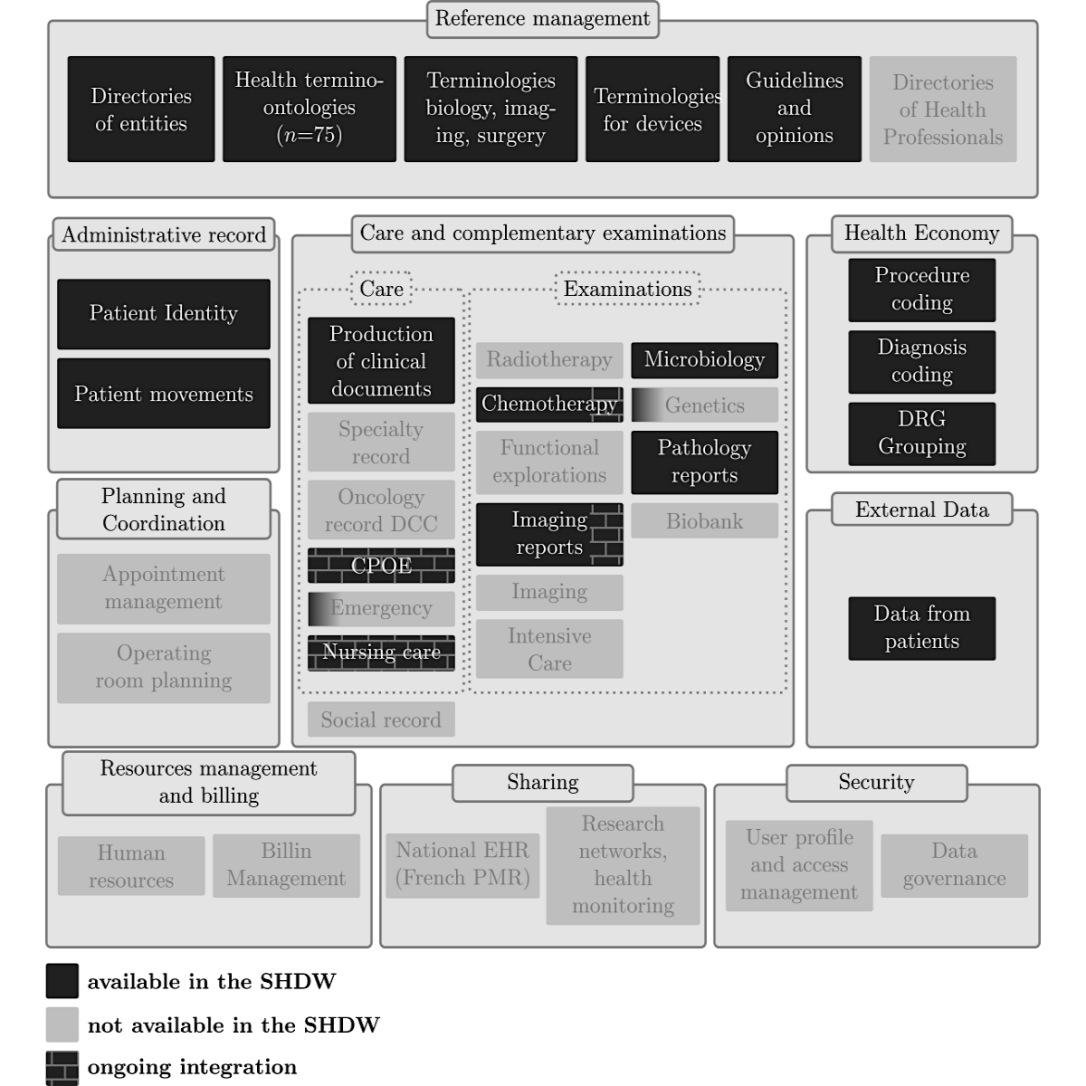
Functional coverage of the semantic health data warehouse in terms of data according to each domain. SHDW: semantic health data warehouse; CPOE: computerized physician order entry; DCC: French cancer communication file; PMR: personal medical record; DRG: diagnosis-related group; EHR: electronic health record.

Since 1992, RUH has collected and maintained patient identity (eg, name, date of birth, and gender), clinical (eg, biological test results, medical procedures, visit records, letters, and discharge summaries), administrative, and less frequently, omics data [34]. The data are produced by different subsystems and applications of the IS of RUH. A subsystem called CPage Dossier Patient partially aggregates some of this important data such as laboratory results, DRGs, procedures, and clinical documents. Other data remain in other subsystems that have to be accessed separately. Overall, RUH maintains the data of 1.8 million patients that represent approximately 14.4 million visits, 11.9 million clinical documents (free-texts recorded since 2000), and 107 million single laboratory tests (eg, Sodium and Potassium being considered as 2 distinct tests; recorded since 2004). Since November 2018, the SHDW POC presented in this study includes the whole set of data. However, this study is based on a randomly chosen subset of data from 207,357 patients, 1.7 million visits, 671,442 clinical documents, and 14.2 million single laboratory tests. ETL scripts are used to incorporate data from the production environment repositories into an Oracle database. Figure 1 summarizes included data according to their specific domain (ie, reference management, administrative record, care, examinations, health economy, planning and coordination, external data, resource management and billing, sharing, and security). Data already included in the semantic health data warehouse (SHDW) are represented by a dark gray opaque background, whereas a light gray background indicates that data are neither included nor planned to be included in the short or medium term. Background partially or totally covered with bricks corresponds to data for which inclusion is in progress or is planned in the short term or medium term.

The SHDW currently focuses on clinical data and, more broadly, on health data according to a patient-centered strategy. In addition to the structured patient data, the different data pertaining to multiple admissions and events at RUH are collected (eg, diagnoses, biology, procedures, and movements). The reference-controlled vocabularies (ie, reference management domain) necessary to the understanding of those data are notably widely collected and maintained. In contrast, pure management and administrative data, such as appointment and planning data, billing data, and data governance, are not likely to be included in the short term. All those data are integrated into a modular architecture that is described in the following section.

### Overall Architecture of the Semantic Health Database Warehouse

**Figure 2:**
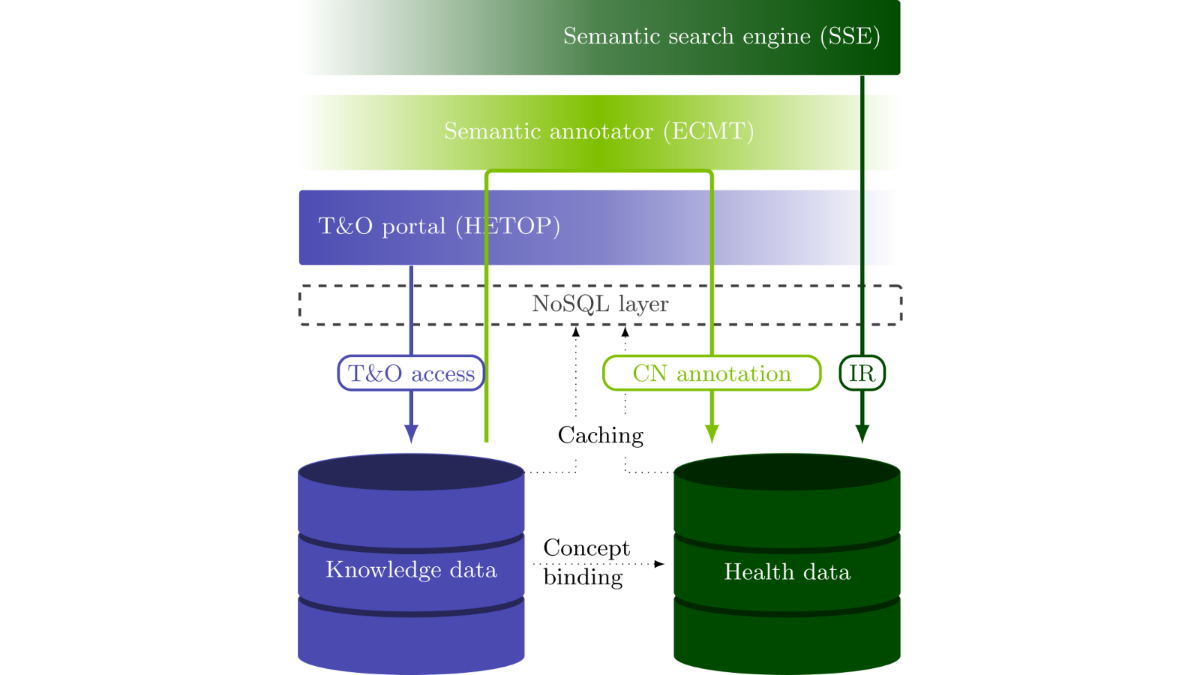
Functional architecture of the semantic health data warehouse that provides semantic information retrieval (IR) functionalities form clinical data. The 2 data repositories, knowledge data and health data, respectively, maintain the reference knowledge organization systems and the health data pertaining to the semantic health data warehouse. These data are accessed through a not only structured query language (NoSQL) layer by the 3 distinct components: the cross-terminological health terminology and ontology portal (HeTOP), the semantic annotator extracting concepts from multiple terminologies (ECMT), and the semantic search engine (SSE), each operating on a different range of data. CN: clinical narrative; T&O: terminology and ontology.

Much health information remains in CNs [7]. The 11.9 million clinical documents in French of RUH consequently play a strategic role in the context of the SHDW. Since its creation in 1995, our research team has strongly investigated French IR research domains through T&Os (and more broadly knowledge organization systems or KOSs), which has led to the development of several search tools mostly dedicated to IR from documentary and bibliographical resources [22,35]. However, the complexity of the clinical data and, more broadly, of SHDWs as a whole required the pooling of several of these acquired skills and tools. The SHDW enables the semantic retrieval of health data in French based on several T&Os and consequently relies on 2 datasets: a domain knowledge database and a health database maintaining clinical and patient data. The functionalities of the SHDW are ensured by the collaboration of 3 distinct layers, where each layer consumes data from the above layers (see Figure 2): the (1) cross-terminological HeTOP [19], (2) semantic annotator ECMT [20,21,36], and (3) SSE [22-24].

The HeTOP provides access to domain knowledge data. The ECMT matches words and expressions in natural language to domain knowledge concepts included in the HeTOP. In fact, ECMT enables the extraction of semantic information from unstructured data. Its functional scope consequently lies between domain knowledge and clinical data. Together, the 2 components HeTOP and ECMT serve as a base for the semantic description of the clinical information in a computer-processable form. In contrast, the SSE and the coupled Web application, are dedicated to IR tasks on health data by using this extracted semantic description.

Considering the amount of data, access to health data and domain knowledge data is made through an NoSQL layer [22] based on the Infinispan solution, an in-memory data grid (IMDG), on a server with 192 cores and 1 TB (ie, 10^12^ bytes) of random access memory (RAM) allowing vertical scaling.

Each of these layers is functionally and technically detailed below.

### Semantic Representation

This section describes data and the methods for data storage and modeling and presents ECMT that enables the link between knowledge data and actual clinical data.

#### Domain Knowledge Data

The HeTOP provides cross-lingual access to concepts originating from 75 T&Os. A set of 2,639,620 concepts and 10,735,905 terms are available mainly in English and French. However, 32 languages are available overall. Some of the T&Os have been partially or totally translated into French (eg, SNOMED 3.5—52.3%; Medical Subject Heading descriptors—100%; National Cancer Institute Thesaurus—53.35%; Online Mendelian Inheritance in Man—79.67%; Human Phenotype Ontology—72.19%; and RadLex—22.1%). More broadly, 50% of the 2.64 million concepts accessible through the HeTOP are provided in French, and 19.1% of the 10.74 million terms have a French translation. T&Os from the HeTOP come with their original sets of hierarchies and semantic relationships but also with additional cross-terminological exact, broader to narrower, and narrower to broader mappings performed manually or supervised by our health professionals at RUH.

As a primary use, the different concepts are bound to the different clinical entities (eg, procedure and DRG codings, and CN annotations), thus allowing a semantic description of the clinical information to be obtained. This allows both refining and broadening of the IR tasks by exploiting the underlying semantic network formed by the concepts (ie, by controlling the granularity and the depth with which this semantic network should be browsed in search processes).

#### Health Data Model

**Figure 3:**
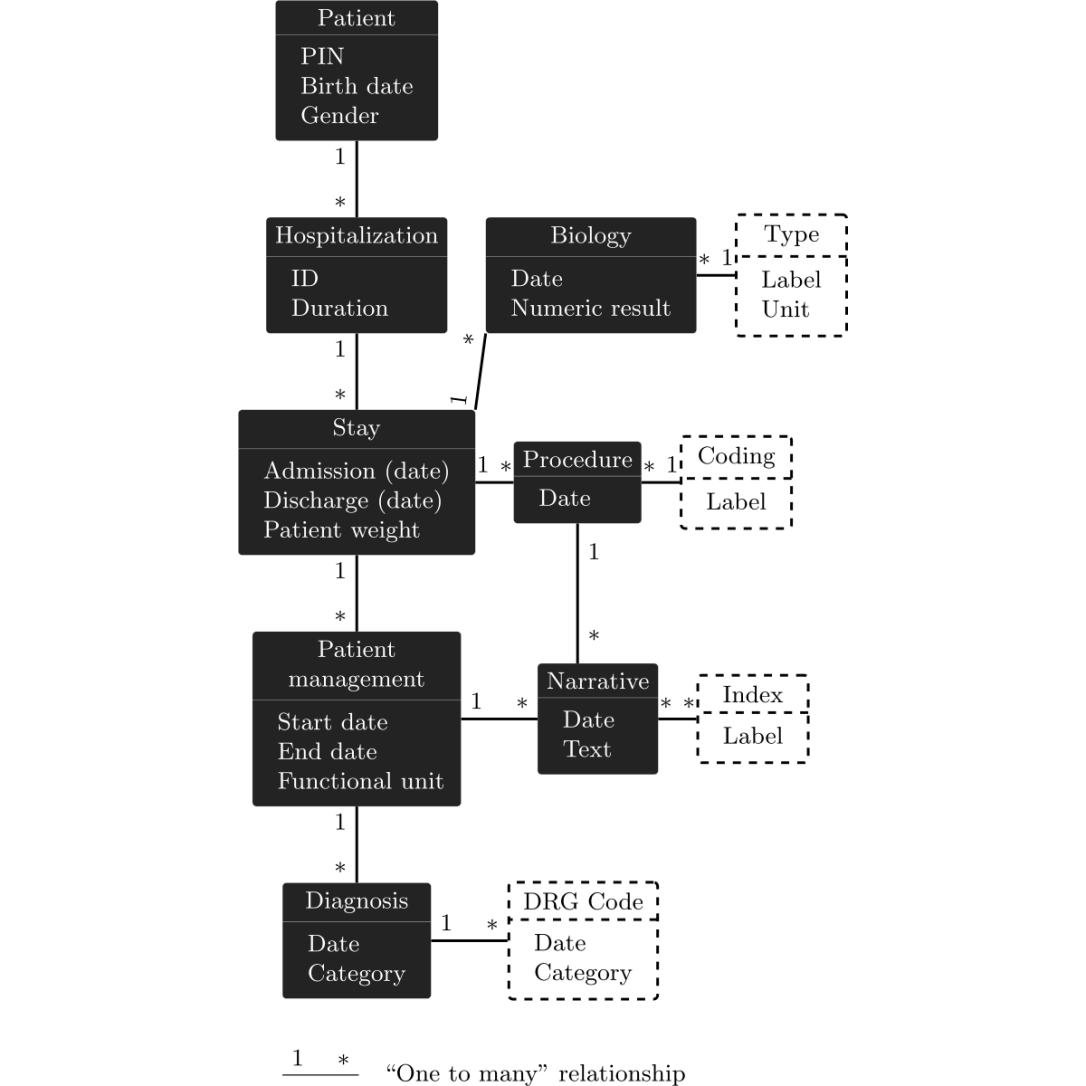
Partial Conceptual Model of the semantic health data warehouse represented as a directed and attributed graph. Entities corresponding to elements from terminologies and ontologies are represented with dashed outlines. DRG: diagnosis-related group; PIN: personal identification number.

Health data are stored in a PostgreSQL [37] relational database. A generic and very adaptable physical EAV data model [34,38] is used to integrate the data. This model structures the information in terms of objects, attributes, and relationships and thus defines an underlying entity-association modeling of the data. It enables the preservation of any original conceptual organization of the information without altering the physical data model and consequently maintains the desired vision of the data at conceptual level. A partial and simplified representation including the main entities and a limited number of relationships and attributes of the conceptual data model used for this study is shown in Figure 3. This model is used on a daily basis to satisfy the information needs of the different health professionals of RUH.

#### Semantic Annotator

The semantic annotator ECMT [20,21,36] matches the natural language words and expressions to the domain knowledge concepts included in the HeTOP. Technically, the ECMT relies on the bag-of-word method for concept matching but also provides pattern-matching functionalities, in particular, to deal with negation and contextual information such as numerical values in CNs. Functionally, the ECMT is used in query building processes to match user inputs to accurate sets of concepts but plays a major role in CN indexing.

### Semantic Retrieval

Access to the data is allowed by a NoSQL layer before processing by the SSE.

#### Not Only Structured Query Language Layer

Owing to the considerable amount of health data that need to be retrieved and the well-known limitations of the RDBMSs in terms of scalability, a NoSQL layer was designed to interface access to all the data and improve data access performances. This layer is based on the IMDG Infinispan [39,40]. It is a Java NoSQL solution that uses key-value hash tables as storage structure, which allows efficient recovery of unitary data via the associated keys. Moreover, the hash tables are stored in memory and not on disk, which leverages access times.

The NoSQL layer was conceived in a generic way to mirror the EAV data model used to structure health data (ie, Java object used as values in hash tables mimics the objects and relationships of the relational databases). This generic NoSQL layer consequently preserves the conceptual data model of health and knowledge data implicitly drawn by the EAV data model. A more detailed description of this layer and an overview of performance gain compared with relational RDBMSs are presented in the study by Lelong et al [22].

#### Semantic Search Engine

The main purpose of the SSE is to deal with the multiplicity and the diversity of conceptual entities inherent to clinical and patient data (eg, patients, stays, CNs, diagnosis, and biological tests). Overall, the entire set of data originating from the SHDW can be seen as a comprehensive oriented attributed graph that can be queried by the SSE. The SSE was designed to concentrate on semantic retrieval by allowing navigation through the semantic networks, not only included in the T&Os but also those representing clinical data conceptual entities. From a more clinically coherent point of view, the data of the SHDW can be organized in 4 levels: (1) patient level corresponding to patient identity information, (2) hospital level defining the sources of information (this level is currently not implemented as all the data originate from RUH), (3) visit level that defines much organizational and administrative information about the health care process, and (4) health level enabling group medical procedures and biological tests [24]. As an HDW can be used in various contexts (eg, health care, health research, and secondary use of health data), access and search capabilities of the full scope of those types of information must be provided. Technically, the SSE is a Boolean and entity-oriented search engine. It enables the retrieval and display of data at any of the previous clinical levels. As mentioned in section above, the NoSQL key-value store used to interface data does not provide proper querying solutions. The SSE consequently relies on a specific query language based on formal grammar. It enables the expression of queries targeting any of the different conceptual entities selected through constraints focusing on attribute values and other linked entities [24]. The SSE is used through a Web application that enables the querying of clinical data using forms and string-based queries. This application is described below.

#### The Semantic Access to a Health Information Web Application: ASIS

**Figure 4:**
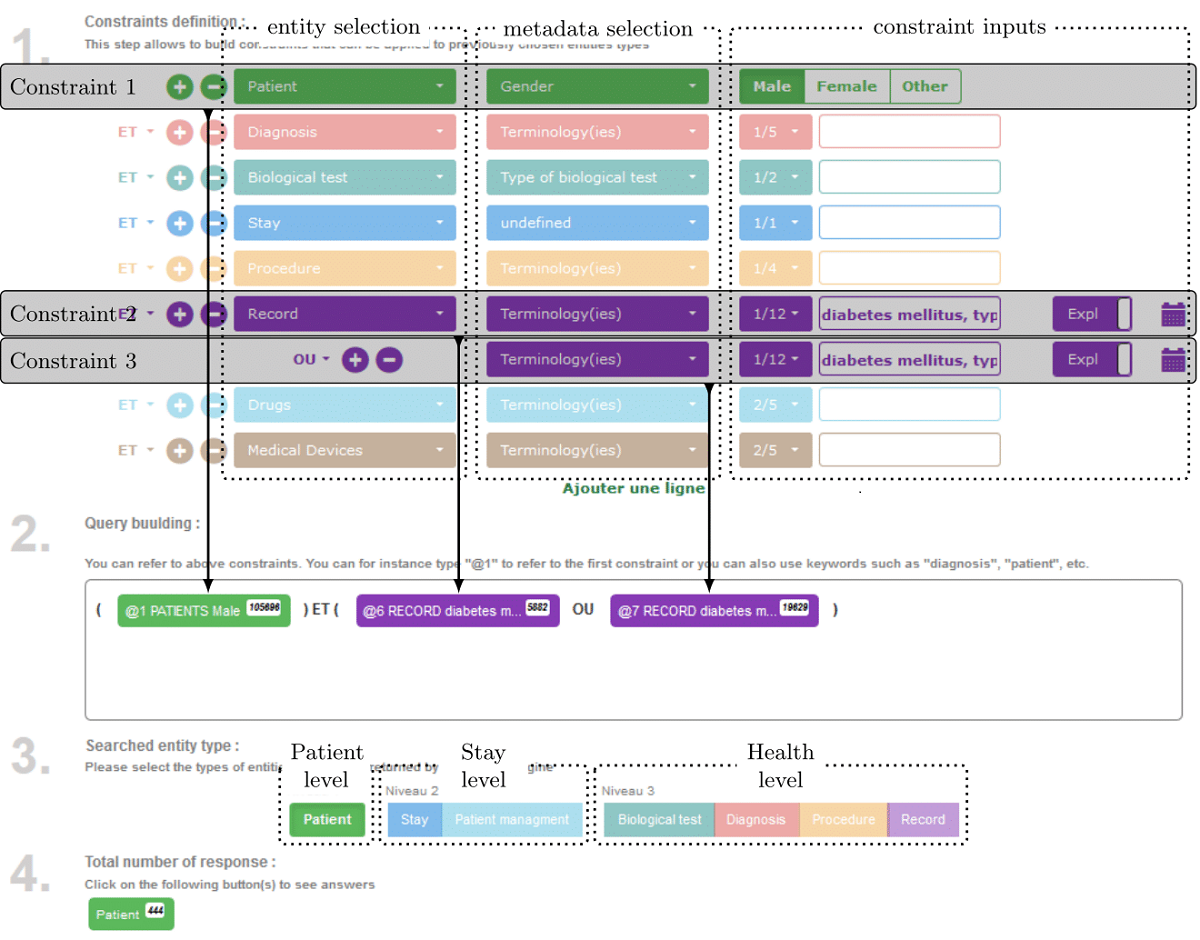
The interface of the semantic access to health information, ASIS, Web application, and its 4 steps: (1) definition of constraints, (2) composition of a Boolean query from atomic constraint defined in step 1, (3) selection of the desired output entity according to its clinical coherent level, and (4) visualization of the results.

The SSE provides a powerful means to select data using textual logical queries. To bypass the complexity of the query language syntax, we designed a user-friendly Web application known as ASIS. It enables the retrieval of clinical data by means of a form that generates an SSE-processable logic-based query. The clinical data selection process is divided into 4 numbered steps clearly identifiable on the graphical interface. Step 1 comprises building a set of constraints related to any desired entity of interest as patient, diagnosis (DRG), biological tests, stays, procedures, records (CNs), drugs, and medical devices (see Figure 4). Constraints are built via (1) the choice of the entity of interest; (2) the choice of the targeted metadata of this entity as date of birth (patient), gender (patient), type of biological test (biological test), date (eg, procedures, biological tests, and stays), and coding (eg, diagnosis, records, and procedures); and finally, (3) the entry of the inputs corresponding to the chosen entity and metadata as male/female for the gender metadata of a patient constraint and the desired numeric value for the biological test constraint. To facilitate the reading of the interface, each type of entity is represented using a specific color (eg, green for patient, red for diagnoses, green-cyan for biology, and blue for stays). As the SHDW was conceived to focus on semantics, many metadata inputs concentrate on selecting T&Os and concepts by the user from fields autocompleted to facilitate the selection. For instance, constraints 2 and 3 enable retrieval of CNs indexed with the different concepts referring to type 1 and 2 diabetes (Figure 4). Step 2 comprises aggregating the constraints defined in step 1 into a Boolean query. In this form, constraints are represented as colored buttons showing their IDs, a short description of them, and the numbers of results of the subqueries corresponding to them. A click on a constraint button enables the visualization of the partial results corresponding to the constraints. The step 2 subform editable area enables the composition of the query using parentheses, Boolean operators (ie, AND, OR, and NOT), and the defined constraints that can be selected using an autocompletion feature. Nevertheless, the step 1 subform enables the predefinition of a basic Boolean query skull that is on-the-fly reported in step 2 and can be later manually modified or left untouched in step 2. Step 3 comprises choosing the desired output entity type classified according to the 3 clinical information levels: patient, visit (stay), and health levels. The choice of an entity type generates a button similar to constraint buttons in step 4.

### Evaluation Methodology

A total of 5 clinical trials originating from the RUH, covering a total of 95 criteria (36 inclusions/59 exclusions), were randomly selected without previous information on the content of those clinical trials. The selection was done from a pool of 57 clinical trials initiated between 2005 and 2018 and that were either still recruiting or already completed. The 5 selected clinical trials were initiated in 2012, 2014, 2015, 2016, and 2018 with various medical objectives. Of them, 3 were still in the recruitment phase. The ability of the system to automate patient prescreening was then assessed on each of those criteria, taken independently from both the originating clinical trial and the overall context of the clinical trials. For each criterion, a search strategy was designed. Each of them required the collaboration of a medical doctor (to clinically interpret the criteria and identify the different sources of information to target) and a computer engineer to master the ASIS tool querying process. The search for a single criterion can be done through multiple search directives (ie, ASIS constraints) targeting different sources of information (ie, entities). Those search directives are then aggregated into a single search strategy (ie, a global query) by combining the different search directives using Boolean operations and relational links between the entities corresponding to each search directive. The different constraints that could reduce the accuracy of each search directive were also investigated. In this study, 3 characteristics are finally considered and linked to each other to more precisely identify the different capabilities and limitations of the system: (1) the global support level of the criteria by the system, (2) the targeted source of information, and (3) the obstacle and barriers that tend to lower the effectiveness of the search.

Each of the criteria was, therefore, classified into 6 levels of global support by the system.

#### Fully Supported Level

This represents the criteria that can be fully automated by the system with a search strategy that retrieves all and only the resources (without false positives or false negatives) that fulfill the exact requirements of the criteria, for example, *18-year-old patients and patients with a neutrophil level below 1700/mm^3^*.

#### Accurately Supported Level

This represents the criteria that are based on consistently recorded data in the IS and on reliable search. The result may, however, possibly include some irrelevant resources or miss relevant ones depending on the choices made in the elaboration process of the search strategy, mainly about the choice of concepts to search and the exploitation of their semantic networks, for example, *patients with hepatitis B or active hepatitis C* and *patient with acute kidney failure*. In absolute terms, a relevant resource may also be ignored if the data have not been entered in a standard way in the IS.

#### Broadly Supported Level

This represents the criteria for which the search results in a lack of precision (ie, inclusion of irrelevant resources or absence of relevant ones). These criteria can only be reliably answered partially. This implies a broadened search of the core requirement of the criteria and a manual postfiltering of the result and/or supervision by a health professional to decide whether, or not, the retrieved resources effectively fit the criteria, for example, *patient with an evolving organic digestive and/or inflammatory pathology* and *patient with a badly regulated cardiac rhythm disturbance*.

#### Inaccurately Supported Level

This represents the criteria that cannot be searched precisely enough (both technically and in terms of data) to fulfill the core requirement of the criteria or systematically provide consistent results. For example, criterion *pregnant woman or breastfeeding mother* cannot be searched correctly as the information used to record pregnancy is only very rarely provided as structured data. Thus, the search for information relating to pregnancy and breastfeeding is, therefore, provided mainly in the reports and is, in addition, not systematically provided. This information is sometime provided in a roundabout way involving an effort of deduction or through hardly analyzable natural language expressions. It is, consequently, a difficult information to retrieve. Nevertheless, this information constitutes the essential part of the medical objective of the criterion. Another type of inaccuracy because of technical limitations of the system can also be observed with criterion *patient admitted for a stomach hemorrhage resulting in a favorable evolution without surgery during the hospitalization*. Although the first part of the criterion is searchable (ie, stomach hemorrhage), the second part of the criterion is not defined with metrics that can be easily interpreted in terms of a query and, more particularly, with regard to *favorable* evolution. In addition, extensive temporal functionalities would have been necessary, in particular, to exclude favorable evolution following surgery.

#### Nonsupported Level

This represents the criteria for which the system fails to properly select the relevant resources (ie, the medical doctor did not consider any of the first results as relevant to the criterion) and/or a search strategy is hardly feasible. For example, the criterion *patient with a regular consumption of licorice or derived substances* is not supported because the patients' diet is an information which is essentially absent from the IS. In the rare cases where the information appears in the unstructured data, this information is incomplete, unreliable, and technically difficult to identify/extract. Similarly, none of the attempts to define a research strategy to solve the criterion *abdominal pain presenting once a week during the last 3 months associated with 2 of the following criteria [...]* yielded consistent results. This criterion involves temporal considerations that are not currently within the scope of the IR system.

#### Not Applicable and Instruction Level

This represents criteria that either does not connect to the medical domain or that corresponds more to instructions than real requirements, for example, *patient participating in another clinical trial* and *contraception will be required during the treatment.*

A total of 6 types of source of information were identified: (P) Patient structured data as age and gender, (D) DRG data corresponding to structured diagnosis coded with the 10th revision of the ICD and related health problems, (S) stay data and other organizational structured data as medical units, (B) biological structured data, (N) CN unstructured data as full-text and/or automatic indexing including drug data, and (I) for information that is not within the scope of RUH IS.

Finally, the different obstacles or limitations that lower the effectiveness of the search were recorded for each atomic search directive and were distributed among 6 categories: (o) for search directives that are free of any obstacles, (d) for data obstacles corresponding to inconsistently provided or insufficiently accurate data from the IS, (s) for difficulties to perform an accurate search in CN or DRG data as complex information search, (t) for technical limitations of the system as chronological querying handling or search for quantitative values in CNs (partially implemented), (c) for subjective and/or generic criteria implying the interpretation or value judgment of a health professional, and (e) when it is necessary to meet the patient to complete the criteria.

The global support levels of criteria observed in this study are first detailed in section *Global Support of Criteria*. A 2-sided Wilcoxon signed-rank statistical test is used to examine the different levels of support of inclusion versus exclusion criteria. The 3 sets of scores detailed in this methodology section are then matched with each other in *Observed Sources of Information and Limitations* to objectify and identify the concrete abilities and limitation of the system.

## Results

### Global Support of Criteria

As a primary and holistic result, the support levels of the 36 inclusion and the 59 exclusion criteria from the 5 randomly selected clinical trials of RUH are shown in Table 1. The percentage of criteria for each of these levels was recorded.

According to the methodology used to classify criteria, 3 out of the 6 levels of support, *full*, *accurate*, and *broad* could be considered as contributing to cohort’s prescreening. Taken together, the system was consequently able, at least partially, to automate the search for 15 out of 36 (15/36, 42%) of inclusion criteria versus 39 out of 59 (39/59, 66%) of exclusion criteria. Among the 5 clinical trials used in this study, the number of exclusion criteria exceeded the number of inclusion criteria by 20% on average (6 vs 16, 9 vs 13, 5 vs 4, 7 vs 13, and 9 vs 13 inclusion vs exclusion criteria, respectively).

**Table 1:** Number, percentage, and 95% confidence interval of the percentage of criteria for each support level and type (inclusion or exclusion).

Support level	Inclusion criteria		Exclusion criteria		Total	
	n (%)	95% CI	n (%)	95% CI	n (%)	95% CI
Full	6 (16.67)	(4.5-28.8)	5 (8.47)	(1.4-15.6)	11 (11.58)	(5.1-18.0)
Accurate	3 (8.33)	(0.0-17.4)	15 (25.42)	(14.3-36.5)	18 (18.95)	(11.1-26.8)
Broad	6 (16.67)	(4.5-28.8)	19 (32.20)	(20.3-44.1)	25 (26.32)	(17.5-35.2)
Inaccurate	4 (11.11)	(0.8-21.4)	6 (10.17)	(2.5-17.9)	10 (10.53)	(4.4-16.7)
None	3 (8.33)	(0.0-17.4)	7 (11.86)	(3.6-20.1)	10 (10.53)	(4.4-16.7)
Not applicable	14 (38.89)	(23.0-54.8)	7 (11.86)	(3.6-20.1)	21 (22.10)	(13.8-30.4)
Total	36 (100.00)	—^a^	59 (100.00)	—	95 (100.00)	—

^a^Not applicable.

A fairer and more reliable measure was also investigated. Of the 95 criteria, 21 (21/95, 22%) of this study were actually not applicable (N/A) criteria. This type of criteria is not in the scope of an HDW-based system and should consequently be set aside. Moreover, 14 of these 21 criteria (67%) were attributed to inclusion criteria. Excluding *N/A* criteria, the percentages of criteria for which the system was able to contribute increased to 68% for inclusions (15/22 criteria) and 75% for exclusions (39/52 criteria). When only considering support levels that did not imply postfiltering (ie, only *full* and *accurate*), 9 out of 22 (9/22, 41%) inclusion criteria could be answered compared with 20 out of 52 (20/52, 38%) exclusion criteria.

A 2-sided Wilcoxon signed-rank statistical test was used to compare the levels of support of inclusion versus exclusion criteria. To perform that test, a mean support score was calculated for each subset of inclusion or exclusion criteria of each clinical trial. The calculation of these means was made by assigning to each support level a score from 0 to 100. The test was not significant with a homogeneous distribution of the scores, but a trend was observed toward better support of inclusion criteria compared with exclusion criteria for distributions that weighted *full* criteria twice as much as the others. The mean support score of inclusion criteria was constantly greater than that of exclusion criteria for each clinical trial. The tests resulted in observed statistics T=15 with a *P* value equal to .06, which, even if slightly greater than the 5% significance level, suggested a better support of inclusion criteria.

### Observed Sources of Information and Limitations

The results obtained in Table 1 should, nevertheless, be regarded more qualitatively than quantitatively as regard the 95% confidence intervals that show widths of 20.37% on average (15.08% when inclusion and exclusion criteria are taken together). To achieve that goal, both the targeted sources of information and the observed limitations for each support level of each criterion were investigated.

The support level of the criteria according to the combination of information sources required to search them are displayed in Table 2.

**Table 2:** Number of criteria of each support level according to the combination of sources necessary to search them. The sources of information are as follows: P: patient data, D: diagnoses-related group data, S: stay data, B: biological data, N: clinical narrative, and I: other information.

Support level	*P*	*S*	*B*	*D*	*N*	*I*	*S+N*	*P+N*	*D+B*	*B+N*	*D+N*	*N+I*	*P+S+D*	*S+D+N*	*D+N+I*
Full	4	0	7	0	0	0	0	0	0	0	0	0	0	0	0
Accurate	1	0	0	8	0	0	0	1	3	0	5	0	0	0	0
Broad	0	2	2	6	7	0	1	0	0	1	4	0	0	2	0
Inaccurate	0	0	0	0	5	0	0	0	0	1	1	0	1	1	1
None	0	0	0	0	2	3	0	0	0	0	3	1	0	1	0
Not applicable	0	0	0	0	0	21	0	0	0	0	0	0	0	0	0
Total	5	2	9	14	14	24	1	1	3	2	13	1	1	4	1

Setting aside N/A criteria, 47 out of the 74 criteria (47/74, 64%) could be answered using a single search directive (ie, by exploiting a single source of information) against 27 criteria (36%) that required combined search directives. The calculation of the mean scores of level of support of these 2 groups of criteria resulted in scores between *accurate* and *broad*. Of single search directives, 23.40% versus 0% of combined search directives concerned fully supported criteria.

The different sources of information were not uniformly distributed. Patients (P) and stays (S) structured data were involved in the search for only 7 out of the 95 (7/95, 7%) and 8 out of the 95 (8/95, 8%) criteria, respectively. In contrast, the top 2 sources of information, CNs (N) and diagnoses (D), were involved in the search of 37 (37/95, 39%) and 36 (36/95, 38%) of the 95 criteria, respectively.

**Figure 5:**
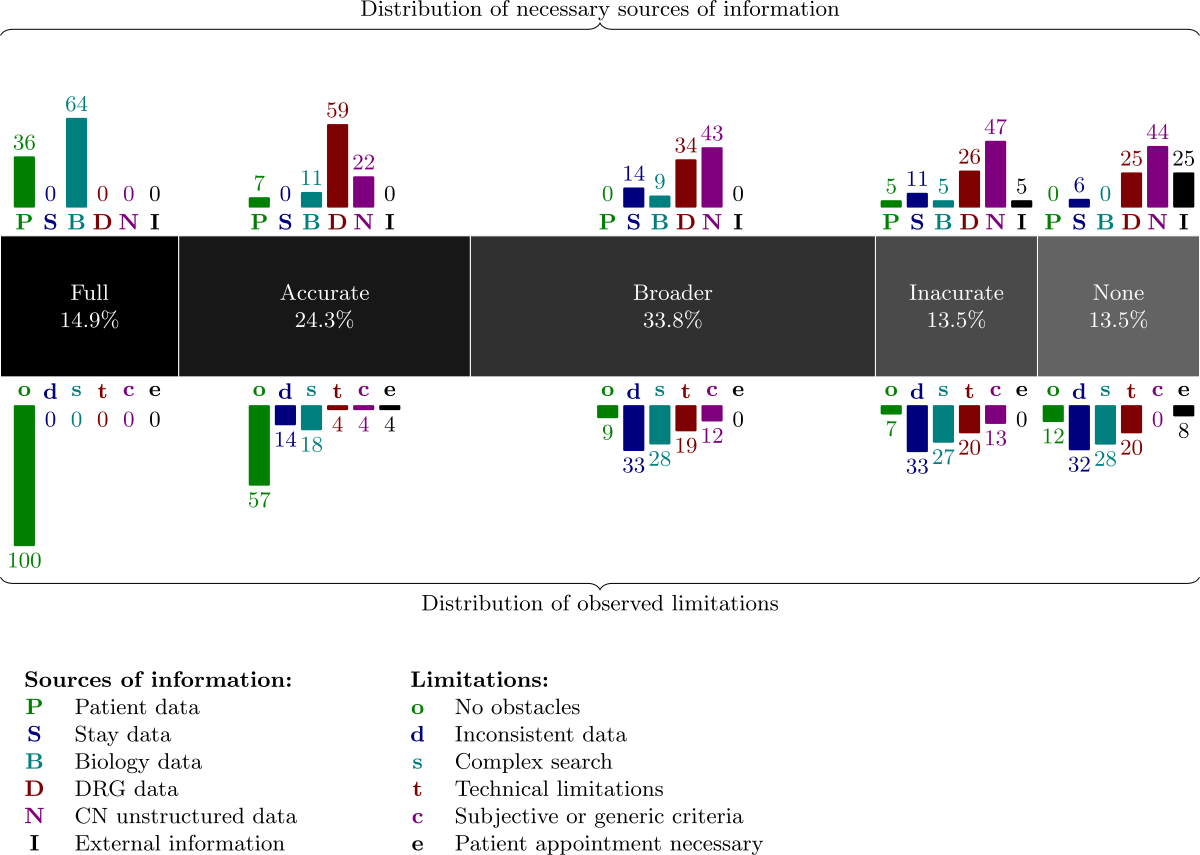
The central gray band gives the percentage of criteria of each support level excluding not applicable criteria. The upper bars show, for each support level, the percentages of involvement of each source of information in the search of criteria. The lower bars show the distribution (in percentage) of the different obstacle categories identified as lowering the effectiveness of the search of criteria. CN: clinical narrative; DRG: diagnosis-related group.

The percentages of involvement of sources of information and the percentages of involvement of observed limitations for each support level are presented in Figure 5.

Only continuously provided and fully structured data were used to answer fully supported criteria. The only sources of information used were patient structured data (P) and biological data (B). Fully supported criteria were consequently based on very precise characteristics not subject to errors or ambiguity and relying on numeric or symbolic data such as *female or male patient aged 18 to 75 years* and *patient with glycated hemoglobin ≤6.5% or ≥8%*.

Accurately supported criteria were mostly searched in DRG data (D). In practice, these criteria either rely on a single source of information (eg, *HIV-positive patient* and *type 2 diabetic subject*) or the combination of data consistently provided or properly coded as “known active hepatopathy, [...], transaminase and/or alkaline phosphatase levels twice the normal level of the laboratory” (DRGs and biological data) or *men aged 18 to 70 years or women aged 18 to 70 years in menopause* (patient data and CNs).

From a holistic point of view, we observed that DRGs (D) and CNs (N) were the 2 major sources of information used. As stated before, both were involved in the search strategy of approximately 38% of criteria (58% if taken together). The support of the criteria by the system decreased as the exploitation of CNs (N) took precedence over DRG data (D). In this study, the search within CNs (N) were performed through both full-text and semantic searching. As far as semantic searching is concerned, the 11,928,168 CNs (N) of RUH have been indexed using ECMT over 55 terminologies available in French from the HeTOP server. These CNs were first collected from the RUH IS in their original format (ie, as Microsoft Word files). The raw text was then extracted from these Word documents and stored in simple text files that were provided as input to the ECMT semantic annotator. After some performance optimizations of this annotator, the indexing of all the CNs could be completed in slightly less than 24 hours on a machine equipped with 1 TB of RAM and 144 cores. The insertion of the annotations in the database was done separately. Indexes allowing retrieval were also generated. The indexing process resulted in a total of 5,043,731,628 annotations. Some of the most redundant concepts were found clinically irrelevant, and a manual filtering process was applied based on the top 5000 most frequent medical concepts (eg, the 27 million annotations with “university hospital” were considered as irrelevant as the information was present elsewhere in the SHDW). A total of 2,087,784,055 annotations were retained after the filtering process. This set of semantic annotations served as a basis for the SSE in the semantic retrieval process.

The search accuracy obstacles (s) category represented 20.2% of all obstacles (32/158), and 84% (27/32) of these obstacles were attributed to CN search (N). However, the exploitation of the semantics (ie, synonyms, hierarchical, and semantic relationships) through the automatic indexing of CNs (N) by the ECMT and the ability of the SSE to combine multiple search directives (using Boolean operators) enabled a broad search support of 25 criteria out of 95 (25/95, 26.3%). Even when postfiltering was required, the system could be used effectively as a prescreening tool. For instance, the search for the criterion *patients with severe heart failure (including New York Heart Association or NYHA Classes III and IV)* was done through the search for *heart failure* in DRG data (D) and the search for *NYHA Classes III* and *IV* in CN data (N). Separately, it resulted in 11,880 diagnoses and 3311 CNs. The combination of both search directives into a single search enabled the prescreening of only 36 patients.

Data inconsistency (d) was also a major challenge, as 37 obstacles out of the 158 obstacles (37/158, 23.4%) were of that type and found across different sources of information including 10 of those (10/37, 27%) for DRGs (D) and 6 of those (6/37, 16%) for stays (S). Many data are sparsely recorded in the IS even outside CNs (eg, weight of the patient as structured data for each stay or S, diet plan in CNs, and hypersensitivity to substances in DRG data or D).

This lack of consistency of information tends to explain the focus on CNs (N) of inaccurately supported (Inaccurate level) and nonsupported (None level) criteria. In practice, these criteria suffer from the association of concurrent obstacles, often including a data consistency obstacle (d). For instance, both data inconsistency (d) and technical limitations (t) were found for the nonsupported criteria *regular consumption of alcohol exceeding 60 g per day*. Information on alcohol consumption was in fact not provided consistently in CNs (N), and technically, it would have required the following: (1) the extraction of a quantitative value from CNs and (2) the processing of this value as data (partially implemented). As another example, the criterion *patient with a creatinine clearance ≤50 ml/mn according to Cockroft formula* was inaccurately managed by searching instead for the biological tests of creatinine higher than 100 μmol/L. The criterion strongly relies on specific calculation functionalities not provided by the system and is based on sparsely provided data (eg, weight of the patient).

With regard to efficiency, the NoSQL layer used to access the data gave querying performances that were considered extremely satisfactory. On the basis of the data of 250,000 patients, each of the search directives used for this study took less than 2 seconds. As far as the POC integrating the entire patient dataset (1.8 million patients) is concerned, similar performances were observed except for some specific queries targeting and returning huge amounts of biological tests which exceeded 1 min.

## Discussion

### Principal Findings

To our knowledge, no formal evaluation of the criteria for clinical trial inclusion and exclusion has been performed using an SHDW. The system based on an SHDW presented in this study could be successfully used to fully automate 29 criteria out of the 74 non-N/A criteria (29/74, 39%). Moreover, with a limited postfiltering process, it could be efficiently used as a prescreening tool for 54 of those (54/74, 73%).

A trend was observed toward better support of inclusion criteria compared with exclusion criteria for distributions of scores that weighted *full* criteria twice as much as the others. However, with homogeneous distributions of scores, no conclusion could be made. A lower support of inclusion requirements tends to affect the ability of the system more to assist prescreening tasks. Manual exclusion of patients is usually a lighter task than manual inclusion (especially if the exclusion is made from a small set of patients who already meet the inclusion criteria). Furthermore, clinical trials usually rely on fewer inclusion than exclusion criteria (36 inclusion vs 59 exclusion criteria in this study), which often makes the inclusion requirements more critical prerequisites. Inclusion criteria are indeed used to target specific medical characteristics essential to clinical trials, whereas some exclusion criteria tend to be more generic. For example, one of the clinical trial used in this study included the specific inclusion criteria *type 2 diabetic subject* and *subject with a weight mass index (weight/height^2^) greater than 27* and, in contrast, more generic inclusion criteria such as *patients with a severe medical or surgical history, in particular, endocrine history* or *patients treated with drugs interfering with the renin-angiotensin-aldosterone system*.

There are still many criteria (ie, 20/74 non-N/A criteria, 27%) that cannot be searched or can only be partially searched by the system. Several mishandled sources of information along with specific limitations of the system are apt to explain these results. DRG and CN data remain an important source of information for nonsupported or inaccurately supported criteria. Consistent and systematic recording of necessary information in the IS not always performed. Furthermore, this information often resides within the unstructured CNs form, which is often difficult to extract.

Technically, our system relies only on free solutions. It accesses the data through an IMDG NoSQL layer that offers very satisfactory performances with the data of 250,000 patients. Since November 2018, all the data of the 1.8 million patients from RUH have been integrated into the POC with relatively constant performance (ie, most of the queries tested in this paper are still under the 5-second threshold considered acceptable by health professionals).

### Comparison to Prior Work

The general philosophy of the system relies on a generic representation of clinical information. It enables the independent search and visualization of each conceptual entity (eg, patient, biology, diagnoses, and CNs) that composes the entire health information of the SHDW. Clinical information originating from HDWs inherits from a complex data structure. This data structure is very different from documentary and bibliographic IR context, which has been studied in the last 2 decades [22,35] and involves a limited number of entities and relationships and where data are classically more *flatly* structured with a limited depth (basically, a resource entity possibly surrounded with several other entities, such as an author or an editor entity). We believe that this entity-oriented vision of the SHDW gives added value to the IR systems dedicated to HDW, compared with existing solutions, such as i2b2, which usually adopt a patient-centered vision and provide the user with aggregated data and lists of patients as a result. Notably, the system allows the search to be conducted in an iterative manner by visualizing the search of each entity before aggregating all of them into a comprehensive and coherent search.

In addition, the underlying powerful query language used by the system makes the querying of entity-based co-occurring events more generic and more intuitive (ie, searching several events occurring in the same stay, hospitalization, and medical units) [23,24]. In contrast, that kind of functionality is usually proposed through user-friendly but predefined and specific forms (eg, STRIDE [14] and i2b2 [25,26]).

One of the fundamental aspects of SHDW is the semantic description of the health information. This was achieved with the help of many health T&Os provided by the HeTOP. The ECMT semantic annotator notably enabled to automatically annotate the 11.9 million CNs, and thus, provided a semantic access to these CNs despite the difficulties to access unstructured information contained within CNs. A bunch of semantic annotators have been proposed for English texts. Recently, Névéol et al [41] performed a literature review on NLP tools in health in languages other than English. In this study, French was the most studied language, followed by German and Chinese. Nevertheless, most of the existing semantic annotators usually extract concepts from the Unified Medical Language System (UMLS) Metathesaurus (eg, MetaMap [12]) [42] or from mainly English T&Os such as the SNOMED-CT terminology (eg, Text Analysis and Knowledge Extraction System, SNOMED-CT and RxNorm [43], and the National Center for Biomedical Ontology Annotator [44]). French is little represented in the UMLS [45]. The HeTOP includes only 17 KOSs of the 2017 edition of the UMLS. However, the UMLS only manages 11 resources providing concepts in French, and among the 978,233 concept unique identifiers of the UMLS included in the HeTOP, only 143,762 (143,762/978,233, 14.7%) concepts in French originate from the UMLS. In contrast, the HeTOP provides access in French to 428,854 of them (428,854/978,233, 43.8%; almost 3 times more than the UMLS).

The vast majority of HDWs are based on RDBMS (eg, i2b2, STRIDE, DW4TR, SMEYEDAT, and Dr Warehouse). In contrast, the system proposed in this study relies on a NoSQL solution that overcomes the limitations of RDBMS as far as the scalability of data is concerned.

### Limitations

From a holistic point of view, the level of support clearly decreased as the CNs became the predominant source of information. The exploitation of unstructured data (N) is consequently considered as the major challenge for the SHDW in this study. More advanced methods of information extraction from those unstructured data, such as the extraction and exploitation of quantitative values from CNs (which is only partially implemented in our system) or the on-the-fly computation of relevant measures (eg, body mass index), could drastically improve the capabilities of the system.

Furthermore, despite the growing interest in statistical machine learning methods, rule-based NLP methods remain predominant as far as clinical information extraction is concerned mainly because of their potential of interoperability and interpretability [9,10]. Nevertheless, since 2018, our team has engaged new research on the semantic annotator ECMT to investigate the development of a hybrid approach between bag-of-words algorithm and word embeddings.

Temporal and chronological aspects are a topic of interest of many IR systems and particularly relevant to IR in clinical data [15]. For instance, DW4TR fully integrates temporal aspects to data modeling by the mean of 3 types of temporal information (eg, static, events, and intervals). Together with clinical and biological data, temporal information is 1 of the 3 dimensions of the 3-dimensional representation of health data in DW4TR. Temporal querying (ie, querying data occurring at a definite moment in time) can be achieved by the underlying search engine of the SHDW and its associated specific query language, but the ASIS Web interface still needs to be enhanced to provide specific forms able to generate the entire set of proper string-based queries. In contrast, the querying of chronologically co-occurring events (ie, searching events occurring before, after, at the same time, or within a definite time frame compared with another) is not well supported. Our department is currently discussing generic technical upgrades of the SSE that will enable us to overcome those limitations and also offer powerful functionalities beyond the scope of time handling.

One of the major drawbacks of NoSQL layer used for data access (ie, Infinispan), and more generally, of many in-memory key-value stores, is that no comprehensive query language is provided as opposed to the SQL for RDBMS. Complex querying capabilities must be fully implemented from the basic application programing interface (ie, obtaining and removing a value of a specific key) proposed by this kind of solution. In particular, in this study, neither join nor reverted index functionality is natively fully provided by Infinispan and requires, respectively, the maintenance of custom maps and the use of Lucene [46] tools to enable the search from concrete values (ie, text and numerical and data values).

An optimized version of the SHDW is, nevertheless, currently in progress.

### Conclusions

An HDW is defined as a grouping of data from diverse sources accessible by a single data management system [13] that centralizes clinical, demographic, and administrative data within a uniform and consistent data model. In this study, a POC of an SHDW based on the data of 250,000 patients from RUH is presented along with a graphical interface semantic access to health information. The system provides semantic IR capabilities and relies on 3 distinct semantic layers. The system was evaluated for its ability to support prescreening of eligible patients in 5 randomly selected clinical trials from RUH. The system showed encouraging results in accurately automating the search of the criteria and good results when used as a prescreening tool. However, this study underlines some limitations of the system especially in relation to information extraction from unstructured CNs, which is still an essential source of information. Since November 2018, all the data of 1.8 million patients from RUH have been included in the POC, and an optimized version is in progress since July 2019.

## Abbreviations

CN: clinical narrative
DRG: diagnosis-related group
DW4TR: Data Warehouse for Translational Research
EAV: entity-attribute-value
ECMT: extracting concepts from multiple terminologies
EMERSE: Electronic Medical Record Search Engine
ETL: extract-transform-load
HDW: health data warehouse
HeTOP: health T&O portal
HIS: Hospital Information System
i2b2: Informatics for Integrating Biology and the Bedside
ICD: International Classification of Diseases
IMDG: in-memory data grid
IR: information retrieval
IS: information system
KOS: knowledge organization system
NLP: natural language processing
NoSQL: not only structured query language
NYHA: New York Heart Association
POC: proof of concept
RAM: random access memory
RDBMS: Relational DataBase Management System
RIM: Reference Information Model
RUH: Rouen University Hospital
SHDW: semantic health data warehouse
SMEYEDAT: SMart Eye DATabase
SNOMED-CT: Systematized Nomenclature Of MEDicine–Clinical Terms
SQL: structured query language
SSE: semantic search engine
STRIDE: Stanford Translational Research Integrated Database Environment
T&O: terminology and ontology
UMLS: Unified Medical Language System
